# Swimming-induced pulmonary edema

**DOI:** 10.36416/1806-3756/e20250501

**Published:** 2026-09-14

**Authors:** Pedro Paulo Teixeira e Silva Torres, Fernanda Miranda de Oliveira, Edson Marchiori

**Affiliations:** 1. Hospital Israelita Albert Einstein, Goiânia (GO) Brasil.; 2. Universidade Federal do Rio de Janeiro, Rio de Janeiro (RJ) Brasil.

A 34-year-old previously healthy man presented with sudden, intense dyspnea associated with pink sputum during a triathlon swimming event. He was referred to the local hospital, arriving with an oxygen saturation of 87%. He received bronchodilators and intravenous corticosteroids, and showed initial clinical improvement. A chest CT scan revealed bilateral ground-glass opacities (Figure 1). Laboratory test results were normal. A respiratory molecular panel was negative. On a subsequent evaluation, the patient was asymptomatic and in good general condition. A follow-up CT scan performed 72 h later was normal.

**Figure 1:**
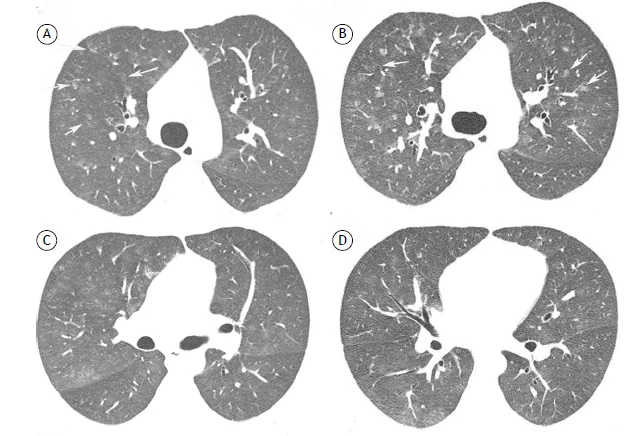
Axial chest CT images showing areas of ground-glass attenuation distributed diffusely throughout the lungs. In A and B, note also ground-glass nodules (arrows).

Swimming-induced pulmonary edema (SIPE), also known as immersion pulmonary edema, is a rare form of non-cardiogenic pulmonary edema that occurs in swimmers. Although the condition is life threatening, its hallmark is the significant resolution of clinical and radiological features within 48 h of treatment initiation. The differentiation of SIPE from post-swimming aspiration pneumonitis is important. By definition, SIPE is associated with pulmonary edema that develops with a closed glottis without drowning or aspiration.^1^ ^-^ ^3^

The risk factors for SIPE include systemic or pulmonary hypertension, underlying cardiac disease, obesity, cold water immersion, heavy exertion, overhydration, female sex, and wetsuit use. The treatment of SIPE consists of the removal of the patient from the water, administration of supplemental oxygen and diuretics, and mechanical ventilation. The prognosis of SIPE is generally good.^1^ ^-^ ^3^
